# Hydrocolloid-Stabilized Magnetite for Efficient Removal of Radioactive Phosphates

**DOI:** 10.1155/2014/504760

**Published:** 2014-02-18

**Authors:** Vinod Vellora Thekkae Padil, Michael Rouha, Miroslav Černík

**Affiliations:** Laboratory of Chemical Remediation Processes, Institute for Nanomaterials, Advanced Technology and Innovation (CXI), Technical University of Liberec, Studentská 1402/2, 461 17 Liberec, Czech Republic

## Abstract

Liquid radioactive waste is a common by-product when using radioactive isotopes in research and medicine. Efficient remediation of such liquid waste is crucial for increasing safety during the necessary storage of the material. Herein, we present a novel Gum Karaya stabilized magnetite for the efficient removal of radioactive phosphorus ^32^P from liquid radioactive waste. This environmentally friendly material is well suited to be used as a nanohydrogel for the removal of liquid waste, which can then be stored in a smaller space and without the risk of the spills inherent to the initial liquid material. The maximum adsorption capacity of the GK/M in this study was found to be 15.68 GBq/g. We present a thorough morphological characterization of the synthesised GK/M, as well as a discussion of the possible phosphorus adsorption mechanisms.

## 1. Introduction

Radioactive waste containing radioactive material is usually a by-product of nuclear power generation and other applications of nuclear technology, such as research and medicine. Radioactive waste is hazardous to most forms of life and the environment. As the radioactivity diminishes over time, the waste is typically isolated and stored for a period of time until it no longer poses a hazard. One of the major approaches to managing radioactive waste has been segregated storage [1]. In the case of liquid radioactive waste, the risk of radioactive spills contaminating a large area, while being stored, is high. Efficient remediation of such liquid waste is therefore crucial for increasing safety during the necessary storage.

Specifically, the radioactive isotope of phosphorus, ^32^P, has been extensively used to trace the path of biochemical reactions. A radioactive tracer can also be used to track the distribution of a substance within a natural system such as a cell or tissue. Radioactive tracers are also used to determine the location of fractures created by hydraulic fracturing in natural gas production. Radioactive tracers also form the basis of a variety of imaging systems, such as PET scans, SPECT scans, and Technetium scans [2]. During the synthesis of ^32^P-labelled biomolecules, and the use of these compounds in various research activities, a large volume of liquid radioactive waste containing phosphates is generated.

There are several available processes used for the removal of nonradioactive phosphates from wastewater, such as reverse osmosis, biological nitrification, and electrodialysis [3–5]. Most of these approaches are, generally, suitable for the removal of high levels of phosphorus contamination; however, they are less suited for low level contamination. Furthermore, most of these methods are relatively expensive; they generate secondary products, such as sludge and foams, and the toxicity of the used materials is not always clear [6]. For low levels of contamination, adsorption is regarded as one of the most effective and economical processes, for which a variety of adsorbents are available [7–9].

Recently, natural gums have been shown to be effective biosorbents for the remediation of toxic heavy metal ions [10–13]. These natural gums are relatively inexpensive, biodegradable, and nontoxic hydrocolloids, which are amenable to both chemical and biochemical modifications [14]. Extensive research has been carried out on Gum Karaya (GK), a partially acetylated polysaccharide produced as an exudate by trees, with a branched structure and a high molecular mass [15]. GK contains about 60% neutral sugars and 40% acidic sugars and is mainly used for food, and nonfood, applications due to its acid stability, high viscosity, and suspension properties [14, 16].

With gum as the adsorbent, magnetic nanoparticles have been successfully used to further improve the efficiency of biosorption by increasing the accessible surface area for the adsorbate [17]. Besides, biomagnetic nanoparticles have great potential in water treatment as their recovery is energy efficient and environmentally friendly. For such an application, it is necessary to use purification methods that do not generate secondary waste and involve only recyclable materials that can be easily used on an industrial scale. Nanoparticles of metal oxides are candidate materials for providing better kinetics for the adsorption of metal ions from aqueous solutions [18, 19].

Specifically, magnetite nanoparticles have attracted increasing research interest in environmental remediation in recent years. Good adsorption activities of magnetite nanoparticles for many heavy metal ions have been reported in the literature [20]. In the water treatment industry, adsorption of phosphate by magnetite has proven to be an effective technique for removing phosphorus from wastewater [21–30]. Magnetite is used not only because of its strong adsorption activity but also because it can be easily separated and collected by an external magnetic field. By this means, it has the capability to treat large amounts of wastewater within a short period of time and then be conveniently separated from the water. Moreover, it can be modified using functionalized polymers and novel molecules to impart surface reactivity.

In this study, we present a novel nontoxic biomagnetic nanoadsorbent, based on surface modification of magnetic iron oxide nanoparticles with Gum Karaya, for the adsorption of radioactive phosphates from liquid radioactive waste. The synthesised Gum Karaya/magnetite (GK/M) nanoparticles are characterised and investigated as an environmentally friendly mediator in the bioremediation of phosphorus. To the best of our knowledge, this is the first time a nanocomposite of a tree-gum and magnetite is used for the bioremediation of radioactive phosphates generated during the synthesis of ^32^P-labelled biomolecules. We also present a detailed study of the adsorption characteristics of the material.

## 2. Experimental Section

### 2.1. Materials

Gum Karaya (GK) powders, FeCl_3_·6H_2_O, FeCl_2_·4H_2_O, and NH_4_OH, were purchased from Sigma-Aldrich (Sigma-Aldrich Company LTD, MO, USA). The ^32^P effluents generated during the synthesis [31] of ^32^P-labelled biomolecules were collected, and the radioactive concentration was determined using liquid scintillation counting (Tri-Carb 2910 TR, Liquid Scintillation Analyser, Perkin Elmer, USA). All chemicals were of analytical grade and used as received without any further purification.

### 2.2. Synthesis of GK/M Nanocomposites

Iron oxide nanoparticles were prepared by coprecipitating Fe^2+^ and Fe^3+^ ions with a NH_4_OH solution and treated under hydrothermal conditions [32]. In detail, that means that 25 mg of GK was suspended in 50 mL Milli-Q-deionized water and agitated at 100 rpm for 15 min to achieve complete dissolution. Another solution containing a mixture of 0.3 M FeCl_2_ and FeCl_3_ (Fe^2+^ : Fe^3+^ = 2 : 1) dissolved in Milli-Q-deionized water was then added to the aqueous gum medium. The whole mixture was kept at ~363 K in a water bath under vigorous stirring. During the reaction process, a suitable amount of NH_4_OH solution was gradually added into the mixture and the pH was maintained above 10. The formation of a black ferrofluidic mixture was observed during the reaction, indicating the formation of iron oxide nanoparticles. Stirring was continued for 30 min. After that, the ferrofluidic mixture was cooled down to room temperature (298.15 K). The resulting GK/M colloidal dispersion was washed several times with Milli-Q water and separated with the help of an external magnetic force. As a last step, the dispersion was freeze-dried to the form of nanocomposites.

### 2.3. Characterization of GK/M Nanocomposites

The composition and morphology of the GK and GK/M nanocomposite were studied using energy dispersive X-ray analysis attached to a scanning electron microscope (ZEISS, Ultra/Plus, Germany). The X-ray diffraction (XRD) pattern of the GK/M nanocomposite was obtained using a Philips X-ray diffractometer with Cu-K*α* radiation (Philips PW 3020, Holland) and the ATR-FTIR of GK and GK/M was obtained on a spectrophotometer (NICOLET IZ10, Thermo Scientific FTIR Spectrometer Company, USA). The spectrometer is equipped with a multireflection variable angle horizontal ATR accessory. Transmission electron microscopy (TEM) analysis of the GK/M colloids was performed using a TEM (Tecnai F 12, Japan) operating at an acceleration voltage of 150 kV. X-ray photoelectron spectroscopy (XPS) analysis of powder samples of GK and GK/M was performed on a Kratos AXIS 165 instrument (Shimadzu Corporation, Kyoto, Japan). XPS spectra were obtained by applying an Mg-K*α* (1253.6 eV) energy source of monochromatic radiation operating at 15 kV and 10 mA. The residual pressure in the analysis chamber was 5 × 10^−10^ Torr (6.66 × 10^−8^ Pa). Survey scans were recorded in the binding energy range 0–1100 eV (1.76239422 × 10^−16^ J) with a pass energy of 80 eV (1.28174125 × 10^−17^ J). Hysteresis loop measurements on the GK/M were performed using a vibrating sample magnetometer (ADE make, model EV9, MicroSense, LLC, Massachusetts, USA) up to field strength of 2 T.

### 2.4. Batch Mode Adsorption Experiments

Biosorption experiments were conducted using solutions having 3.7 TBq/m^3^ of ^32^P with an optimum GK/M concentration (1000 g/m^3^). The solutions (100 mL) including the GK/M were shaken for the desired contact time in an electrically thermostatic reciprocating shaker (Innova-43, New Brunswick Scientific Co. LTD, NJ, USA) at 200 rpm. Contact time, optimum pH, initial radioactive phosphate concentration, sorbent dose, and adsorption capacity experiments were performed at room temperature (298 ± 2) K. The batch studies were performed at different experimental conditions such as initial radioactive phosphorus concentration (0.37–3.7 TBq/m^3^), contact time (10–180 min), pH (2–10), GK/M concentration (0.1–5.0 kg/m^3^), and temperature (298–328 K). The equilibrium time was estimated by drawing samples at regular intervals until equilibrium was reached. The adsorption (%) of ^32^P and adsorption capacity (*q*_*e*_) of GK/M were calculated. The experiments were run in triplicate; the mean of the results obtained is used in the analysis.

### 2.5. Langmuir and Freundlich Adsorption Isotherm Models

The sorption isotherms of the ^32^P on GK/M were determined based on batch experiments at (298 ± 2) K. The GK/M nanocomposite (100 g/m^3^) was allowed to equilibrate with solutions at different initial ^32^P concentrations (0.37–3.7 TBq/m^3^). The pH value of the solutions was adjusted to (6.0 ± 0.1). The whole mixture was kept at a contact time of 120 min (equilibration time) in an electrically thermostatic reciprocating shaker (Innova-43, New Brunswick Scientific Co., LTD, NJ, USA) at 200 rpm. After the equilibration time, the mixture was centrifuged for 10 min at 5000 rpm. The ^32^P concentration in the supernatant was measured using liquid scintillation counting (Tri-Carb 2910 TR, Liquid Scintillation Analyser, Perkin Elmer, USA).

In order to optimize the biosorption process parameter, both Langmuir and Freundlich isotherm models were tested on the experimental results [33, 34]. The Langmuir model, which is based on the assumption that maximum adsorption occurs when a saturated monolayer of solute molecules is present on the adsorbent surface, the energy of adsorption being a constant and with no migration of adsorbed molecules in the surface plane, is given by

(1) $$\begin{array}{c} q_{e}=\frac{q_{\max}k_{l}C_{e}}{1+k_{l}C_{e}}, \end{array}$$

where *q*_*e*_ and *q*_max⁡_ are the equilibrium and maximum uptake capacities, *C*_*e*_ is the equilibrium concentration, and *k*_*l*_ is the Langmuir isotherm constant related to free energy of adsorption. The slope of the plot of *C*_*e*_/*q*_*e*_ against *C*_*e*_ gives 1/*q*_*m*_, the intercept 1/*q*_max⁡_ *k*_*l*_.

The Freundlich isotherm model is given by the equation

(2) $$\begin{array}{c} q_{e}=k_{F}{C_{e}}^{1/n}, \end{array}$$

where *k*_*F*_ and *n* are Freundlich's constants related to the adsorption capacity and intensity of adsorption, respectively. The values of *k*_*F*_ and *n* can be determined from the log-log plot of *q*_*e*_ versus *C*_*e*_ as the intercept and slope, respectively.

### 2.6. Kinetic Studies

In order to examine the controlling mechanism of the adsorption process, kinetic models are used to test the experimental data. The kinetic adsorption data can be processed to understand the dynamics of the adsorption reaction in terms of the order of the rate constant. Pseudo-first-order and pseudo-second-order kinetic models may present the process of ^32^P adsorption by the GK/M nanocomposite from the radioactive effluents. Conformity between the experimental data and the model-predicted values is expressed by the correlation coefficient (*R* ^2^). The closer its value is to unity, the more accurately the model describes the kinetics of adsorption.

The pseudo-first-order kinetic model describes the adsorption rate based on the adsorption capacity. Its linear form is generally expressed as

(3) $$\begin{array}{c} \log\left(q_{e}-q_{t}\right)=\log q_{e}-\frac{k_{1}}{2.303}t, \end{array}$$

where *q*_*e*_ and *q*_*t*_ are the adsorption capacity and adsorption at time *t*, respectively, and *k*_1_ is the first order rate constant. A plot of log (*q*_*e*_ − *q*_*t*_) versus *t* gives *k*_1_ as the slope and log *q*_*e*_ as the intercept value.

The pseudo-second-order model applied to adsorption kinetics was given by Ho and McKay [35] as

(4) $$\begin{array}{c} \frac{t}{q_{t}}=\frac{1}{k_{2}q_{e}^{2}}+\frac{1}{q_{e}}t, \end{array}$$

where *q*_*t*_ is the amount of sorbate on the sorbent at time *t*, *k*_2_ is the rate constant of pseudo-second-order sorption kinetics, and *q*_*e*_ is the equilibrium uptake. The plot of *t*/*q*_*t*_ versus *t* should show a linear relationship, if the second-order kinetics are applicable. Values of *k*_2_ and *q*_*e*_ were calculated from the intercept and slope of the plots.

### 2.7. Thermodynamics of Adsorption

The energy of activation for the adsorption processes was calculated by using the Arrhenius equation, and other thermodynamic parameters such as change in free energy, enthalpy, and entropy associated with the adsorption process. The energetics of the adsorption process was also examined by temperature studies. The temperature dependency on the rate of a reaction is important to predict the feasibility of the reaction. Arrhenius has shown the relation between the rate constant and the temperature as follows:

(5) $$\begin{array}{c} \ln k_{2}=\ln A-\frac{E_{A}}{RT}, \end{array}$$

where *k*_2_ is the rate constant, *A* is the Arrhenius factor, *R* is the universal gas constant, and *T* is the thermodynamic temperature. The value of the activation energy *E*_*a*_ is determined from the slope of ln⁡⁡*k*_2_ versus 1/*T*.

The thermodynamic aspects of ^32^P ions adsorption by the GK/M nanocomposite were investigated using thermodynamic parameters such as enthalpy change (Δ*H* ^0^), free energy change (Δ*G* ^0^), and entropy change (Δ*S* ^0^). The free energy change of the adsorption reaction is given by the following equation:

(6) $$\begin{array}{c} \Delta G^{0}=-RT\ln K_{D}, \end{array}$$

where Δ*G* ^0^ is the free energy change, *R* is the universal gas constant, *T* is the thermodynamic temperature, and *K*_*D*_ (*q*_*e*_/*C*_*e*_) is the distribution coefficient.

According to the Van't Hoff equation:

(7) $$\begin{array}{c} \ln K_{D}=\frac{\Delta S^{0}}{R}-\frac{\Delta H^{0}}{RT}, \end{array}$$

the enthalpy change (Δ*H* ^0^) and the entropy change (Δ*S* ^0^) parameters can be calculated. Δ*H* ^0^ and Δ*S* ^0^ values were calculated from the slope and intercept of the plot of ln⁡⁡*K*_*D*_ versus 1/*T*, respectively.

## 3. Results and Discussion

### 3.1. Morphological Characterization

**Figure 1:**
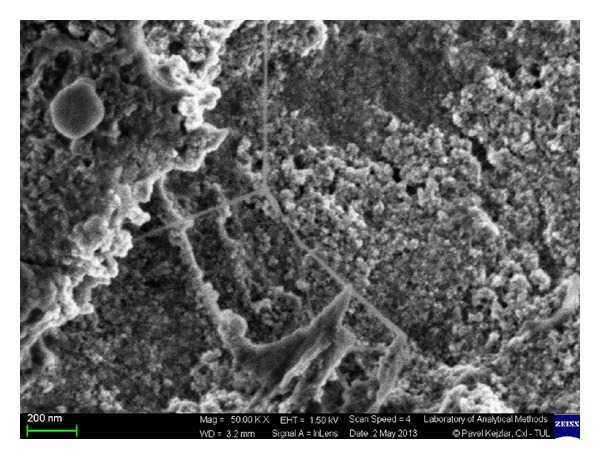
Scanning electron micrograph image of the GK/M nanocomposite, showing the morphological changes due to the formation of magnetite.

**Figure 2:**
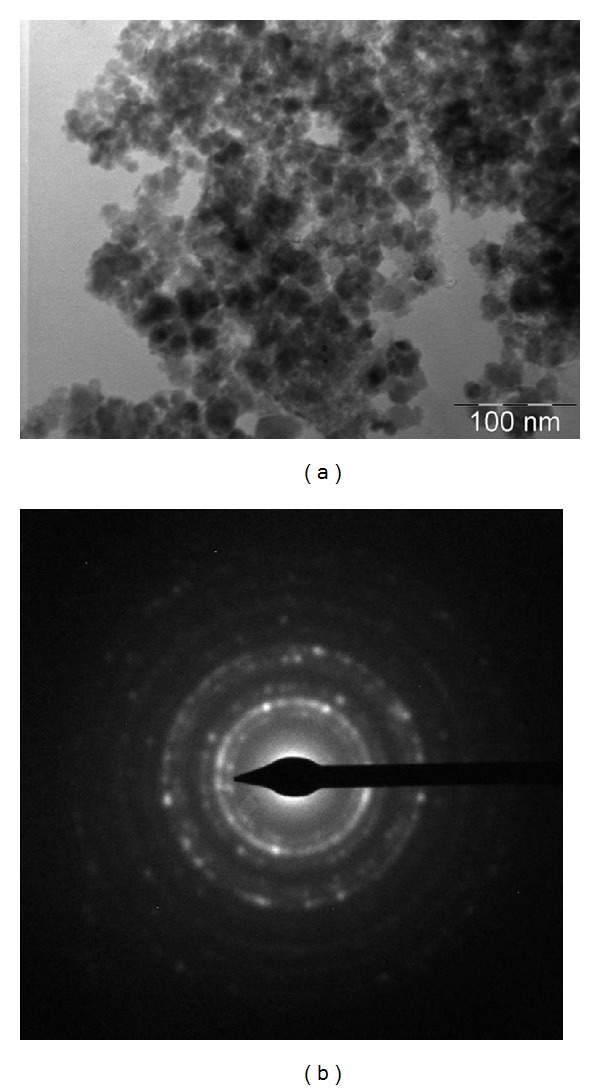
Transmission electron microscope image of the GK/M nanocomposite showing the formation of magnetite nanoparticles (a) and the corresponding SAED pattern (b).

To investigate the surface and particle size distribution of the nanoparticles formed during the chemical process, the morphology of the synthesised GK/M nanocomposite was characterised and compared to that of pure GK. The scanning electron micrograph of GK/M (Figure 1) clearly shows the magnetite beads before phosphate adsorption. Upon the functionalization of the iron oxide nanoparticles, the branched-network morphology of the GK has been significantly modified into spherical aggregate structures containing iron oxide nanoparticles, as illustrated by the TEM micrograph (Figure 2). The size of the iron oxide nanoparticles within the gum matrix was in the range of 10–15 nm.

**Figure 3:**
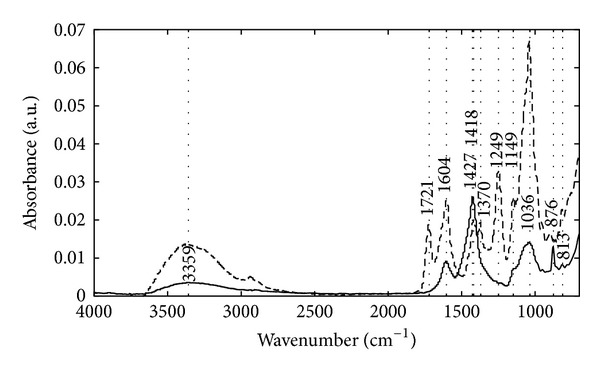
Fourier transform infrared spectroscopy analysis of pure Gum Karaya (dotted line) and the GK/M nanocomposite (full line), indicating the involvement of various functional groups of GK interacted with Magnetite to form GK/M nanocomposite.

FT-IR characterization was carried out to identify the functional groups that are responsible for the formation of the iron nanoparticles treated with GK. Due to the binding of the iron oxide nanoparticles to the surface of the GK matrix, the intensity of the FT-IR spectra peak at 1036 cm^−1^ is considerably contracted in the case of the GK/M nanocomposite (Figure 3). The intensity of the O–H stretching at 3,359 cm^−1^ for the GK/M is lower than that of the native gum. The peaks at 1,721 cm^−1^ and 1,249 cm^−1^ (CH_3_CO group) present in the GK are displaced in the GK/M samples, which suggests that the acetyl groups play an important role in the formation of the GK/M nanocomposite. Another peak shift, from 1,427 cm^−1^ to 1,370 cm^−1^, is observed in the case of GK/M, implying that both the carbonyl and hydroxyl functional groups of GK have a strong affinity to bind the magnetite nanoparticles. The lower peak at 1,604 cm^−1^ in the GK/M, compared to the peak for GK, indicates that the negatively charged COO groups bind to positive sites in the surface of the magnetite.

**Figure 4:**
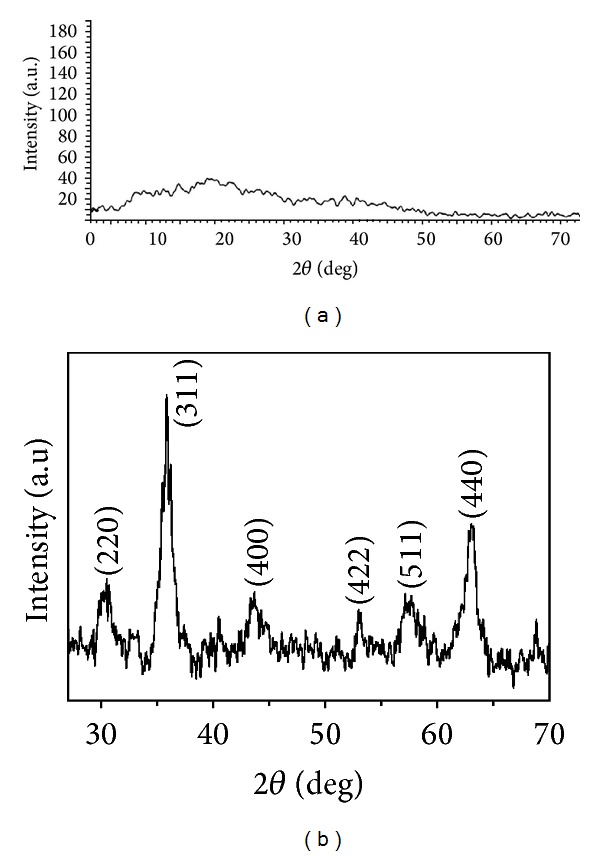
X-ray diffraction pattern of (a) GK, showing an amorphous nature, and (b) GK/M, showing crystalline structures.

XRD analysis was performed to evaluate the formation of the GK/M nanocomposite. The amorphous nature of GK can be seen from the XRD patterns (Figure 4(a)), which have no intense peaks. GK/M on the other hand (Figure 4(b)), has distinct peaks corresponding to the formation of iron oxide nanoparticles within the network of the GK matrix. The diffraction peaks at 30.4°, 35.9°, 43.5°, 52.9°, 57.3°, and 62.8° correspond to the (220), (311), (400), (422), (511), and (440) planes of bulk crystalline magnetite (JCPDS number 75-1610), respectively. The XRD pattern thus clearly shows that the synthesised iron oxide nanoparticles are crystalline in nature. A broadening of peaks due to the small crystal size effect can be seen in the case of GK/M. The crystallite size, calculated using the Debye-Scherer formula [36], is ~(11.0 ± 5.0) nm, which is in good agreement with the aforementioned range of 10–15 nm obtained by the TEM analysis.

**Figure 5:**
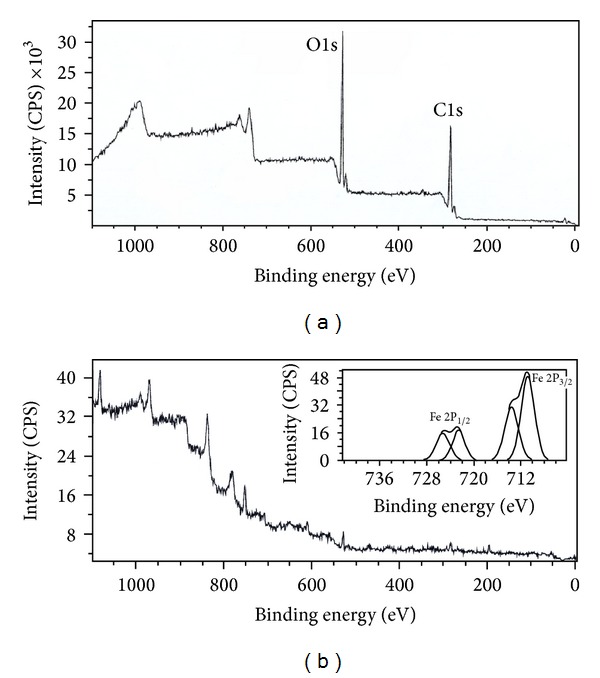
X-ray photoelectron spectroscopy of (a) pure GK, showing the major oxygen (1s) and carbon (1s) peaks, and (b) GK/M nanocomposite. The spectrum together with spectra of Fe (2P_3/2_, 2P_1/2_) (inset) is presented. The peak at ~715 eV corresponds to Fe 2P_3/2_ of Fe^3+^ and the small peak at ~723 eV corresponds to Fe 2P_1/2_, confirming the formation of magnetite.

We used XPS to further confirm the presence of magnetite and find its oxidation state. The XPS survey scan (Figure 5), shows the binding energy peaks of carbon and oxygen in GK and in the case of GK/M the Fe2p spectrum for magnetite. The 2P_3/2_ peak is centred at binding energy 710.2 eV (1.1378658 × 10^−16^ J), and the 2P_1/2_ peak is at 732.8 eV (1.17407499 × 10^−16^ J), which matches well with the reference data of magnetite [37].

**Figure 6:**
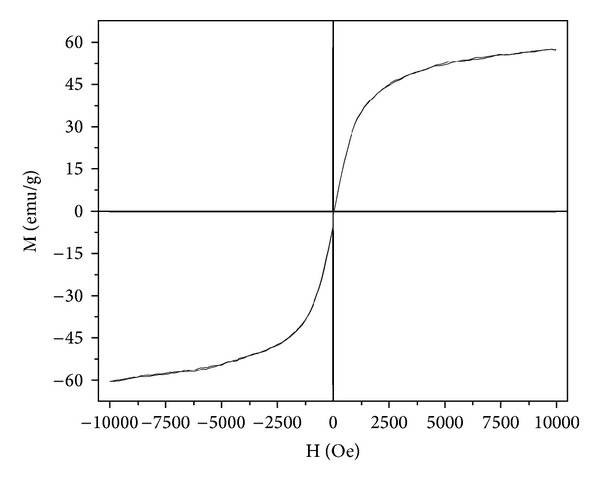
Hysteresis loop of the GK/M nanocomposite showing the relation between the applied magnetic field, H, and the magnetization, M.

The magnetic properties of GK/M were investigated using a vibrating sample magnetometer at room temperature with an applied field ranging from −398 to 398 A/m. The hysteresis loop of the GK/M nanocomposite (Figure 6) shows ferromagnetic behaviour with magnetic saturation values of ~60 m^2^A/kg. The magnetization values indicate that GK/M can have a strong magnetic response and therefore can easily be separated from the solution with the help of an external magnetic force. This magnetization value of GK/M is comparable to earlier reported values for magnetic chitosan nanocomposites [38], another natural product, showing that GK/M might have the same potential for applications as the widely used chitosan.

### 3.2. Adsorption Study

**Figure 7:**
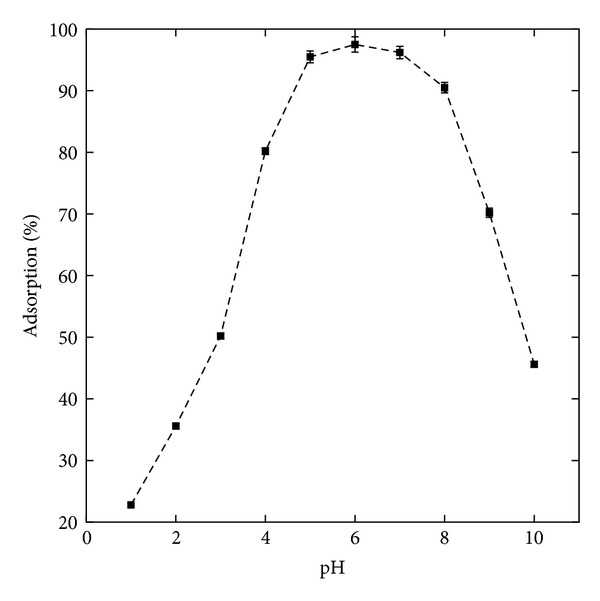
Adsorption characteristic of the studied GK/M nanocomposite, showing the effect of the initial pH on the adsorption of ^32^P.

The maximum adsorption (%) of ^32^P, obtained from adsorption experiments conducted at different pH (1–10) (Figure 7) was found to be at pH 5–7 and reaches (96.4 ± 1.0)%. Fe(OH)_3_ is found in a hydrated form and precipitates as an amorphous complex that changes its structure over time. The mechanisms responsible for this are ion exchange and precipitation of ^32^P on the surface of the GK/M nanocomposite. It was suggested [39] that phosphates form bonds with the metal hydroxyl complex. These bonds are very strong and bind the phosphate to the structure. It was previously reported that the stoichiometric ratio of metal and phosphorus in the precipitant depends on many factors, including the phosphate concentration in the liquid, the chemical dose, the age of the hydroxyl complex, and mixing [40, 41].

Our previous work on pure Gum Karaya [15] indicates that the formation of complexes with phosphorous is favoured due to the high amount of calcium present in the gum. It was previously reported [30] that calcium ions can form stable complexes with phosphorus and that a high amount of calcium enhances the retention capacity of phosphorus irrespective of pH. Several studies have shown that Ca^2+^ was effective for the removal of phosphorus from an aqueous solution by surface precipitation. Magnetite surface was used as nucleus for heterogeneous Ca-phosphate precipitation [42, 43]. It was earlier reported that phosphate adsorbed on magnetite by inner-sphere complex mechanism [44].

**Figure 8:**
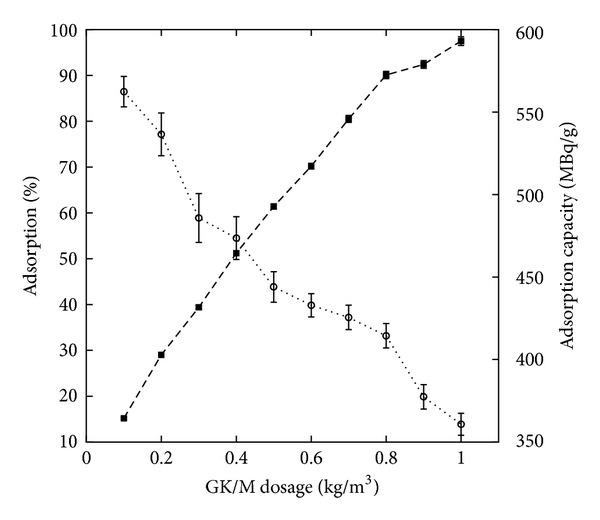
Adsorption characteristic of the studied GK/M nanocomposite, showing the effect of GK/M nanocomposite dosage on the adsorption (squares), and the effect on the adsorption capacity of ^32^P (circles).

**Figure 9:**
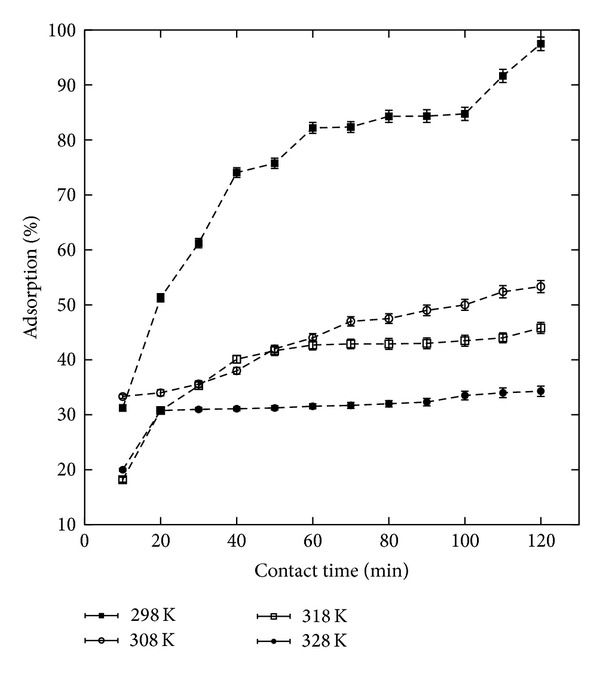
Adsorption characteristic of the studied GK/M nanocomposite at various temperatures, showing the effect of contact time on the adsorption of ^32^P.

We further investigated the adsorption capacity for ^32^P as a function of GK/M nanocomposite dosage (Figure 8). The removal of ^32^P was found to be dependent on the concentration of the GK/M nanocomposite. We find the expected result [13] that with an increase in the nanocomposite concentration the metal removal efficiency is enhanced due to an increase in the number of adsorption sites. On the other hand, with an increase of the GK/M nanocomposite concentration from 0.1 to 1 kg/m^3^, the adsorption capacity decreased from (562.4 ± 9.25) to (360.75 ± 6.66) MBq/g (Figure 8). Increasing the concentration further has no effect on the adsorption, as saturation is achieved. The drop in adsorption capacity is due to an excess of adsorption sites that due to a lack of material remain unsaturated during the adsorption process. The effect of the contact time on ^32^P adsorption on the GK/M nanocomposite was also studied (Figure 9) and we observed an increase in the adsorption capacity of ^32^P by the GK/M nanocomposite with an increase in the contact time. The initial rapid adsorption changes to a very slow process and the equilibrium point is reached after 120 min. The nature of the adsorbent and its available sorption sites affected the time needed to reach equilibrium.

**Figure 10:**
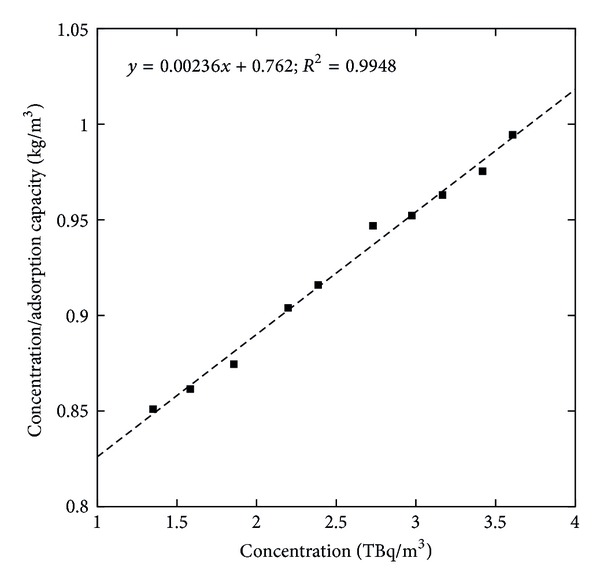
Langmuir adsorption isotherms for the adsorption of ^32^P onto the GK/M nanocomposite.

**Figure 11:**
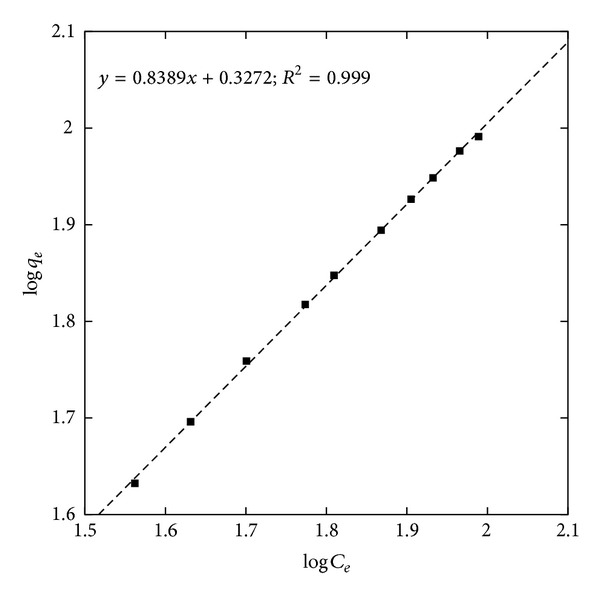
Freundlich adsorption isotherms for the adsorption of ^32^P onto the GK/M nanocomposite.

To reveal the specific relation between the concentration of the adsorbate and the adsorption capacity of the adsorbent at a constant temperature, we interpreted the experimental equilibrium adsorption data using nonlinear forms of the Langmuir and Freundlich isotherm models. The Langmuir isotherms for adsorption of ^32^P on GK/M at optimized experimental conditions (Figure 10) give the maximum amount of ^32^P adsorbed to be 15.68 GBq/g. The adsorption energy is 3 × 10^−6^ m^3^/g. The nonlinear Freundlich isotherm (Figure 11) giving *k*_*F*_ = 2.12 and *n* = 1.19, also satisfactorily describes the relationship between the amount of ^32^P adsorbed by the nanocomposite and its equilibrium concentration in the solution.

We employed the Lagergren first-order kinetic model to describe the adsorption rate based on the adsorption capacity. The first-order kinetic model parameters (Table 1) do not fit the experimental results satisfactorily; therefore, the calculated adsorption capacity *q*_*e*_ does not agree with the experimental values. This is an indication that the biosorption of ^32^P by the GK/M nanocomposite does not follow the pseudo-first-order kinetic reaction.

The pseudo-second-order model, based on the sorption capacity of a solid phase, was then used to fit the results. The second-order sorption rate constant (*k*_2_) and adsorption capacity (*q*_*e*_) values (Table 1) were determined from the slopes and intercepts of the plots of *t*/*q*_*t*_ versus *t*. The *q*_*e*_ values calculated by the model agree perfectly with the experimental values. This suggests that the biosorption follows the pseudo-second-order model. This model is based on the assumption that the rate-limiting step may be a chemical sorption involving valence forces through sharing or exchange of electrons between the adsorbent and the adsorbate [45]. This provides the best correlation for the experimental data.

We further examined the energetics of the adsorption process by thermodynamic studies. The temperature dependence on the rate of reaction is important to predict the feasibility of the reaction. It can be described by a simple but remarkably accurate formula, Arrhenius' equation. When evaluated, the activation energy is *E*_*a*_ = 82.1 kJ/mol, and the preexponential factor is *A* = 0.0427 m^3^/mol/s. From the value of the activation energy, we can derive whether the adsorption mechanism is diffusion-controlled (*E*_*a*_ < 42 kJ/mol) or surface-controlled (*E*_*a*_ > 42 kJ/mol) [46]. The activation energy therefore clearly indicates that the adsorption of ^32^P on the GK/M nanocomposite is a chemical precipitation process, that is, a surface-controlled reaction.

The thermodynamic aspects of ^32^P ion adsorption by the GK/M nanocomposite were further investigated based on the equation in Section 2.7. The Gibbs free energy change (Δ*G* ^0^) for the adsorption of the ^32^P ions on the GK/M nanocomposite was found to be −10.49 kJ/mol at a temperature of 298 K, −10.84 kJ/mol at 308 K, −11.19 kJ/mol at 318 K, and −11.34 kJ/mol at 328 K. The negative value of the Gibbs free energy change indicates that the biosorption is thermodynamically feasible and of a spontaneous nature. Furthermore, the decrease of the Gibbs free energy change with increasing temperature shows a decrease in the feasibility of the biosorption.

In the temperature range of 298.15–328.15 K at an initial pH of 6 and initial ^32^P concentration of 3.7 TBq/m^3^, the enthalpy change is Δ*H* ^0^ = 23.32 J/mol and the entropy change is Δ*S* ^0^ = 35.28 J/mol/K. The positive value of the enthalpy change shows that the biosorption of ^32^P ions on the GK/M nanocomposite is endothermic in nature; that is, the *K*_*D*_ values decrease with increasing temperature. The positive value of Δ*S* ^0^ suggests some structural changes in the adsorbate and the adsorbent during the adsorption process [47].

### 3.3. Storage of ^32^P Effluents

After adsorption of ^32^P by the GK/M, the excess aqueous phase was removed from the mixture using a speed Vac concentrator. The ^32^P content was also determined using liquid scintillation and no radioactive contamination was found in the aqueous phase. The ^32^P adsorbed by the GK/M was stored until a negligible level of radioactive contamination was detected in this nano-composite. The results indicated that the GK/M could be developed as a green biosorbent for effective storage of radioactive isotopes and it could also help to reduce the amount of liquid radioactive waste in a gel form, in order to save precious water.

### 3.4. Desorption

In order to apply biosorbent to real industrial effluent samples, desorption process of toxic material is essential.Almost 98% of the adsorbed ^32^P was desorbed from the GK/M nanogel using 0.1 N HCl as an eluent. The release of ^32^P from the GK/M by acid suggests an ion-exchange mechanism. The adsorption-desorption cycle was repeated five times with the same adsorbent using 0.1 N HCl. The adsorption capacity with the regeneration cycles varied from 98% to 90% from one to five cycles of operation.

## 4. Conclusions

In this work, we synthesised and characterised a Karaya hydrocolloid stabilized magnetite. This composite of gum and magnetite was then successfully used for the removal of radioactive phosphates generated from ^32^P-labelled biomolecules. To the best of our knowledge, no other material for the removal of radioactive phosphorus has been presented so far. The presented study shows that the synthesised magnetite effectively removes radioactive phosphorus, especially at low concentrations of input phosphorus, with a maximum adsorption capacity of 15.68 GBq/g. The contact and sedimentation times of the presented remediation method are also relatively short, which is a desirable property for real-life application. Based on our findings, the possibility arises to fabricate nontoxic hydrocolloid-based magnetic nanohydrogels that could be used for the long-term storage of initially liquid radioactive waste. The results for phosphorus further suggest that the studied biodegradable hydrocolloid-based materials could be used for the efficient remediation of other radionuclides. The presented material also represents a green alternative to common methods of nonradioactive phosphate removal.

## Figures and Tables

**Table 1:** Kinetic studies of ^32^P adsorption onto the GK/M nanocomposite. Showing experimental (EXP), pseudo-first-order kinetic model (KM1), pseudo-second-order kinetic model (KM2) values of the equilibrium uptake capacity *q*_*e*_, and model related quantities (Section 2.5). Units: *q*_*e*_ (GBq/g), *k*_1_ (1/min), and *k*_2_ (g/TBq/min).

*T* (K)	EXP	KM1			KM2		
	*q*_*e*_	*k*_1_ × 10^2^	*q*_*e*_	*R* ^2^	*k*_2_ × 10^−3^	*q*_*e*_	*R* ^2^
298.15	**3.842**	1.98	**2.395**	0.907	1.308	**3.608**	0.99
308.15	**1.973**	2.11	**1.121**	0.967	0.092	**2.009**	0.99
318.15	**1.695**	2.43	**0.767**	0.855	0.053	**1.793**	0.99
328.15	**1.268**	1.63	**0.279**	0.55	0.026	**1.333**	0.99
